# Recombinant Chimeric Transmissible Gastroenteritis Virus (TGEV)—Porcine Epidemic Diarrhea Virus (PEDV) Virus Provides Protection against Virulent PEDV

**DOI:** 10.3390/v11080682

**Published:** 2019-07-25

**Authors:** Alejandro Pascual-Iglesias, Carlos M. Sanchez, Zoltan Penzes, Isabel Sola, Luis Enjuanes, Sonia Zuñiga

**Affiliations:** 1 Department of Molecular and Cell Biology, National Center of Biotechnology (CNB-CSIC), Campus Universidad Autónoma de Madrid, Darwin 3, 28049 Madrid, Spain; 2 Ceva Animal Health, Ceva-Phylaxia, Szallas u. 5, 1107 Budapest, Hungary

**Keywords:** PEDV, modified live-vaccine, attenuated virus, protection, enteric virus

## Abstract

Porcine epidemic diarrhea virus (PEDV) is an enteric coronavirus causing high morbidity and mortality in porcine herds worldwide. Although both inactivated and live attenuated vaccines have been extensively used, the emergence of highly virulent strains and the recurrent outbreaks even in vaccinated farms highlight the need of effective vaccines. Engineering of genetically defined live attenuated vaccines is a rational approach for novel vaccine development. In this line, we engineered an attenuated virus based on the transmissible gastroenteritis virus (TGEV) genome, expressing a chimeric spike protein from a virulent United States (US) PEDV strain. This virus (rTGEV-RS-SPEDV) was attenuated in highly-sensitive five-day-old piglets, as infected animals did not lose weight and none of them died. In addition, the virus caused very minor tissue damage compared with a virulent virus. The rTGEV-RS-SPEDV vaccine candidate was also attenuated in three-week-old animals that were used to evaluate the protection conferred by this virus, compared with the protection induced by infection with a virulent PEDV US strain (PEDV-NVSL). The rTGEV-RS-SPEDV virus protected against challenge with a virulent PEDV strain, reducing challenge virus titers in jejunum and leading to undetectable challenge virus RNA levels in feces. The rTGEV-RS-SPEDV virus induced a humoral immune response specific for PEDV, including neutralizing antibodies. Altogether, the data indicated that rTGEV-RS-SPEDV is a promising vaccine candidate against virulent PEDV infection.

## 1. Introduction

Acute infectious diarrhea is a major cause of high morbidity and mortality in piglets worldwide. Enteric infections in animals are frequently associated with viruses, including rotaviruses and coronaviruses (CoVs) [1]. A metagenomics analysis of diarrheic and healthy samples from China in 2012 found porcine CoVs in 77% of the diarrheic samples, and only in around 7% of the healthy samples, highlighting the potential relevance of CoVs as enteric porcine pathogens [2]. Porcine epidemic diarrhea virus (PEDV) was found in more than 50% of the diarrheic samples [2], in agreement with the importance that this virus has for the porcine industry worldwide. PEDV was first described in Europe in the 1970s and the virus has, since then, remained endemic in the European porcine herds [3]. In Asian countries, PEDV was detected in the 1980s, with highly virulent outbreaks observed since 2010 [4]. In 2013, virulent strains related to those circulating in China were detected in the United States (US). Due to the absence of pre-existing immunity, PEDV spread very fast in the US, with around 50% of the herds infected in one year [5]. As a consequence of the high mortality and morbidity caused by the disease, PEDV has generated very important economic losses in the US, i.e., after the first outbreak the loss was estimated as $1 billion [6], and recently estimated as $432 per sow [7].

PEDV is classified within the subgenus *Pedacovirus* of genus *Alphacoronavirus*, included in the family *Coronaviridae* into the order *Nidovirales* [8,9]. CoVs contain the largest known genome among RNA viruses, consisting of a single-stranded, positive-sense, 5′-capped and polyadenylated RNA molecule that, in the case of PEDV, is around 28 kb in length [10]. PEDV mainly infects small intestinal villous enterocytes, causing acute necrosis that leads to villi atrophy [11]. This could produce severe diarrhea as a consequence of malabsorption. Often, diarrhea is accompanied by vomiting, which increases severe dehydration and anorexia. These clinical signs could lead to animal death. The severity of clinical signs caused by PEDV is inversely related to the age of animals. Clinical signs are very severe in nursing piglets, under two-weeks of age, with a mortality rate of up to 95%. In weaned to finisher pigs and pregnant sows, the clinical signs are milder and self-limiting within five to 10 days after the onset of disease [12].

Both inactivated and live attenuated PEDV vaccines are manufactured and have been extensively used in Asia (http://www.cfsph.iastate.edu/Vaccines/). The emergence of virulent PEDV strains in China during 2010, even in previously vaccinated farms, and in 2013 in the US points to the need for more effective vaccines. Therefore, PEDV vaccine development has been focused in the application of previously used strategies to obtain vaccine candidates derived from novel strains [13,14,15,16]. Subunit or inactivated vaccines were quickly developed, as their design and generation used to be fast and easy. Nevertheless, they have been proved non-efficient to control PEDV [16,17,18,19,20,21,22]. These vaccines are only partially effective in the induction of mucosal immunity, although they have been proposed for boosting immunity in sows prior to farrowing [23,24]. Vaccination with live virus is the best strategy to induce lactogenic immunity and protection against PEDV infection of newborn piglets [25,26]. Due to the rapid spread of PEDV in the US, some veterinarians have recommended intentional infection of sows during gestation to induce lactogenic immunity (feed-back approach). It was rapidly assumed that, once a herd was infected, long-term immunity would be developed. Apart from potential risks following intentional exposure to PEDV, it leads to decreased lactogenic immune responses in herds with pre-existing immunity, being one of the less effective tools for PEDV elimination in that case [1,27]. In fact, re-infections have been frequently reported in farms applying the feed-back approach, even sometimes produced by the same PEDV strain that originally infected the herd [26,28]. On the other hand, current live attenuated vaccines did not prevent virus shedding. This could represent a biosafety problem as the vaccine strain can revert to virulence by recombination with circulating strains. In fact, such recombinant PEDV isolates have been identified in Asia [29]. Therefore, novel effective and safe vaccines against PEDV are required.

The engineering of well-defined recombinant enteric porcine CoVs may represent major progress towards the development of more effective and biosafe vaccines. To this end, a combination of reverse genetics systems, using infectious cDNA clones, and introduction of attenuating modifications are required. The first CoV infectious cDNA clone was engineered by our group for transmissible gastroenteritis virus (TGEV), as a bacterial artificial chromosome (BAC) [30]. Spike (S) protein is the major structural protein in CoV envelope and is involved in receptor binding and membrane fusion [31,32,33,34]. By modifying S protein sequence, CoV tropism is altered [35,36,37,38] (C.M. Sanchez, A. Pascual-Iglesias, S. Zuñiga and L. Enjuanes, manuscript in preparation). Moreover, species tropism could also be changed by introducing S sequences in the genomes of distantly related CoVs [39,40,41,42]. In addition, S protein is the main inducer of neutralizing antibodies and, indeed, many CoV vaccines are based in the expression of S protein or S protein domains [23,43,44,45].

In this work, we engineered a chimeric recombinant TGEV virus (rTGEV), expressing the ectodomain of a virulent US PEDV S protein. The chimeric virus was viable and initially virulent in piglets. Subsequently, an attenuated chimeric vaccine candidate was developed by introducing duplication of transcription regulating sequences (TRSs), as previously described by our group [46]. This attenuated vaccine candidate conferred partial protection to pigs from a challenge with a virulent PEDV.

## 2. Materials and Methods

### 2.1. Ethics Statement

Experiments involving animals were performed in strict accordance with EU (2010/63/UE) and Spanish (RD 1201/2005 and 32/2007) guidelines. The procedures were performed at Prophyl Ltd. (Mohacs, Hungary). All the protocols were approved by the in site Institutional Animal Welfare Committee (IAWC) on February 8th 2017. The National Scientific Ethical Committee on Animal Experimentation approved the protocols on February 28th 2017 [reference number: BA02/2000-19/2017 (KA-2122)].

### 2.2. Cells and Viruses

Baby hamster kidney cells stably transformed with the gene coding for porcine aminopeptidase N (BHK-pAPN) [47] were grown in in Dulbecco’s modified Eagle’s medium (DMEM) supplemented with 5% fetal calf serum (FCS) and G418 (1.5 mg/mL) as selection agent. Vero cells were grown in DMEM supplemented with heat-inactivated 10% FCS. A US virulent PEDV strain (PEDV-NVSL, GenBank accession number KF267450) was kindly provided by Ceva Animal Health. A recombinant virulent US PEDV virus (rPEDV), recovered from an infectious cDNA engineered by our group (A. Pascual-Iglesias, L. Enjuanes and S. Zuñiga, unpublished data), was used as challenge virus in the animal experiments. PEDV infectious cDNA was maintained as a BAC, including PEDV USA/Iowa/18984/2013 sequence (GenBank accession number KF804028). PEDV viruses and recombinant TGEV viruses obtained in this work were grown in Vero cells supplemented with infection medium (DMEM supplemented with 0.3% tryptose phosphate broth, 0.02% yeast extract, and 10 µg/mL trypsin). The culture medium was supplied daily with 80% of the initial trypsin amount.

### 2.3. Plasmid Constructs

A DNA fragment containing the S gene sequence from virulent PEDV USA/Iowa/18984/2013 (GenBank accession number KF804028) [48] was chemically synthesized and purchased from GeneArt (Regensburg, Germany). A pGEM-T plasmid containing nucleotides 20,287 to 24,811 of TGEV-SC11 genome (GenBank accession AJ271965) and engineered PacI and MluI restriction sites [46] were used as an intermediate plasmid. Overlapping polymerase chain reaction (PCR) was used to fuse PEDV and TGEV S gene sequences. The PEDV S sequence encoding the ectodomain was amplified using purchased S sequence as template and oligonucleotides Pac-S-VS (5′-GGA*TTAATTAA*GAAGGGTAAGTTGCTCATTAGAAATAATGGTAAGTTACTAAACTTTGGTAACCACTTCGTTAACACACCATGAAGTCTTTAACCTAC-3′, PacI restriction site in italics) and PEDV-S-3952-RS (5′-CCACACATACCAAGGCCACTTGATGTATGTCTC-3′). TGEV sequence encoding S protein transmembrane domain and endodomain was amplified using pGEM-T-TGEV-SC11 intermediate plasmid as a template and oligonucleotides TGEV-S-4165-VS (5′-GAGACATACATCAAGTGGCCTTGGTATGTGTGG-3′) and TGEV-S-4439-RS (5′-TCCA*ACGCGT*AAGTTTAG-3′, MluI restriction site in italics). In all cases, PEDV sequences are indicated underlined. The obtained 4073 bp and 308 bp PCR products were used as templates and amplified with oligonucleotides Pac-S-VS and TGEV-S-4439-RS. The 4348 bp PCR product was digested with PacI and MluI and cloned into the same sites of pGEM-T-TGEV-SC11 plasmid, generating pGEM-TGEV-SPEDV plasmid. This plasmid was digested with PacI and MluI restriction enzymes and the fragment containing chimeric S gene was cloned into the same sites of pBAC-TGEV-SC11-P-M or pBAC-TGEV-SC11-RS [46]. All cloning steps were checked by sequencing of the PCR fragments and cloning junctions. For each mutant sequence, two independent cDNAs were constructed.

### 2.4. Transfection and Recovery of Infectious rTGEVs from cDNA Clones

BHK-pAPN cells grown to 90% confluence in 35 mm plates were transfected using 4 µg of the corresponding pBAC and 12 μL of Lipofectamine 2000 (Invitrogen, Carlsbad, CA, USA), according to manufacturer’s specifications. At 6 h post-transfection (hpt), BHK-pAPN transfected cells were trypsinized, washed twice with DMEM, and plated over confluent Vero monolayers grown in 35-mm-diameter plates. One hour later, trypsin was added to a final concentration of 10 µg/mL. After a 3-day incubation period, the cell supernatants were harvested (passage 0) [49]. The rTGEVs were cloned by limiting dilution method, grown, and titrated as previously described [50].

### 2.5. Analysis of rTGEVs Virulence

Twenty-one five-day-old suckling piglets, born from TGEV and PEDV seronegative sows, were randomly divided in two groups of nine piglets and one group of three animals. The nine-piglet groups were orally inoculated with 10^6^ TCID_50_/animal of the different rTGEVs in phosphate buffered saline (PBS). The three piglets in the other group were mock inoculated. Infected animals were monitored daily to detect signs of enteric disease, and body weights were determined each day. Three animals per group were euthanized and necropsied at days 2, 4, and 6 after inoculation. Intestinal macroscopic lesions were evaluated. Front, mid, and end sections of jejunum were collected in all cases. Samples were kept frozen for subsequent virus titration, stabilized with RNAlater stabilization reagent (Life Technologies, Carlsbad, CA, USA) for RNA extraction, or fixed in Zinc Formaline fixative (Sigma-Aldrich, St. Louis, MO, USA) for histopathology.

### 2.6. Intestinal Damage Measurement

Fixed jejunum sections were washed twice with PBS and stored in 70% ethanol at 4 °C. Paraffin embedding, sectioning, and hematoxylin-eosin staining were performed by the histological laboratory (Autopsy Path Kft., Budapest, Hungary). Samples were examined with a ZEISS Axiophot fluorescence microscope. Determination of the jejunum damage was obtained from unbiased measurement of three full-length perceived villi and crypt per location. At least two different locations per section per animal were measured. The villous height to crypt depth (VH/CD) ratio was calculated from the measurements.

### 2.7. Immunization of Piglets

Twenty-one 21-day-old piglets, born from TGEV and PEDV seronegative sows, were randomly divided in four groups (Table 1). Piglets of groups 1 and 2 were orally inoculated with 10^6^ TCID_50_/animal of rTGEV-RS-SPEDV or PEDV-NVSL, respectively. Piglets of groups 3 and 4 were mock inoculated. Three weeks after the immunization, piglets of groups 1, 2, and 3 were challenged with 10^7^ TCID_50_ of rPEDV per animal by oral route. Infected animals were monitored daily to detect signs of enteric disease, and body weights were determined every 7 days. Fecal swabs were collected at days 0, 7, 21, 25, 28, and 31 post-first inoculation. Serum samples were taken at days 0, 21, 28, and 31 post-immunization. Saliva samples were collected, using Salivette tubes (Sarstedt, Nümbrecht, Germany), at days 21, 28, and 31 post-first inoculation. Three animals per group were euthanized and necropsied at days 4 and 10 post-challenge (25 and 31 post-immunization, respectively). Intestinal macroscopic lesions were evaluated. Front, mid, and end sections of jejunum were collected in all cases. Samples were kept frozen for subsequent virus titration, stabilized with RNAlater stabilization reagent (Life Technologies) for RNA extraction, or fixed in Zinc Formaline fixative (Sigma-Aldrich) for histopathology.

### 2.8. Enzyme-Linked Immunosorbent Assay (ELISA)

Antibodies induced against TGEV and PEDV viruses were detected by ELISA as described before [51]. Briefly, ELISAs were performed using 0.75 μg per well of partially purified TGEV, PEDV-NVSL, or rPEDV viruses. Antigens were bound to 96-well microplates, saturated with 5% bovine serum albumin (BSA) in PBS for 2 h at 37 °C, and incubated with serial dilutions of the serum sample in Wash Buffer (0.1% BSA, 0.05% Tween20 in PBS) for 90 min at 37 °C. Microplates were washed three times with Wash Buffer. Bound antibodies were detected by incubation with peroxidase-conjugated protein A, goat anti pig IgG (Fc domain), or goat anti pig IgA (BioRad, Hercules, CA, USA), diluted 1:10,000 in PBS with 0.1% BSA. ELISA was developed with K-Blue TMB substrate (Neogen, Lexington, KY, USA) for 5 min at room temperature. Reactions were stopped with 1.5 M H_2_SO_4_, and the absorbance was read at 450 nm. The ELISA values of the sera were expressed as sample to positive ratio [SP-ratio = (OD of sample − OD of negative control)/(OD of positive control − OD of negative control)].

### 2.9. Neutralization Assay

Heat-inactivated sera were incubated for 1 h at 37 °C in the presence of 100 pfus of PEDV virus in DMEM containing 5% FCS. Serial dilutions of the mixtures were added to confluent Vero cells in 96-well plates. After one hour of incubation at 37 °C, medium was removed and infection medium was added. After 72 h, cells were fixed with 10% formaldehyde in PBS, stained with a crystal violet solution, and virus titer was determined. The neutralization index was defined as the logarithm of the ratio of TCID_50_ in the presence of medium or in the presence of serum sample.

### 2.10. Analysis of Viral RNA

Total intracellular RNA was extracted from Vero cells or tissue samples. Total RNA was purified with RNeasy Mini kit (Qiagen, Hilden, Germany), according to the manufacturer’s specifications. To isolate viral RNA from fecal swabs, the swab was resuspended in 300 µL of PBS with antibiotics (40 U/mL penicillin-streptomycin, 200 µg/mL gentamicin, 1 µg/mL amphotericin B). RNA was isolated from 140 µL of that solution using QIAamp Viral Mini kit (Qiagen) following the manufacturers’ instructions. In all cases, total cDNA was synthesized using 100 ng of total RNA as a template, random hexamers, and the High-capacity cDNA transcription kit (Life Technologies), following the manufacturers’ recommendations.

The viral genome region from 3b gene to 3′ UTR (nt. 25856–28544) was sequenced in all cases. To that end, overlapping PCRs were performed using oligo pairs described in Table 2. The PCR fragments were then sequenced using specific oligonucleotides.

PEDV genomic RNA (gRNA) levels were measured by RT-qPCR analysis using a custom TaqMan assay detecting a conserved ORF1a region (TaqMan Probe 6-FAM-TGTACTGGCTTACTGGTGTT-MGB; forward primer 5′-TGTTGCTATGTTTGTGCATTGG-3′; reverse primer 5′-TCTGAATCACTAGGCTGACCTTTG-3′). TGEV gRNA was evaluated using a custom TaqMan assay set up in our laboratory [52]. The porcine β-glucuronidase (GUSB) gene (TaqMan code Ss03387751_u1) was used as a reference housekeeping gene, since its expression remains constant in infected cells compared to that in non-infected cells [[53] and data not shown]. Data were acquired with a 7500 real-time PCR system (Applied Biosystems, Foster City, CA, USA) and analyzed with 7500 software v2.0.6. The relative quantifications were performed using the 2^−ΔΔ*Ct*^ method [54]. All experiments and data analysis were MIQE (Minimum Information for publication of Quantitative real-time PCR Experiments guidelines) compliant [55].

### 2.11. Statistics Analysis

Two-tailed, unpaired Student *t* tests were used to analyze the difference in mean values between groups. All results were expressed as means ± the standard deviations of the means. *p* values <0.05 were considered significant.

## 3. Results

### 3.1. Engineering of TGEV Infectious cDNAs Expressing S Protein from a Virulent PEDV

**Figure 1:**
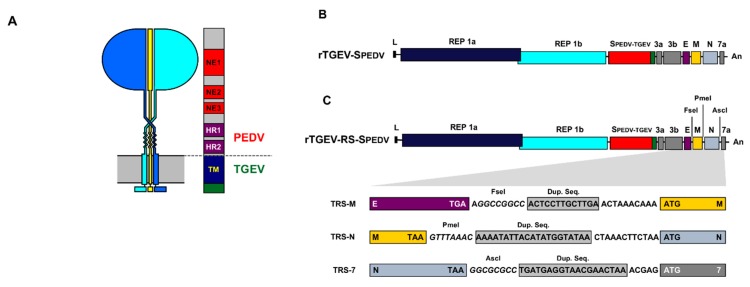
Generation of rTGEV-SPEDV viruses. (**A**) Diagram of the chimeric spike (S) protein expressed by the virus. A representation of spike peplomer is indicated in the left. The scheme on the right indicates the S protein sequence from porcine epidemic diarrhea virus (PEDV) and from TGEV. Relevant S protein domains are indicated: NE, domains recognized by neutralizing antibodies; HR, heptad repeat domain; TM, transmembrane domain. (**B**) Scheme of the recombinant TGEV virus genome encoding a chimeric S protein (SPEDV-TGEV). Labels above the boxes indicate viral genes. L, leader sequence; An, poly A tail. (**C**) Scheme of the rTGEV virus expressing the chimeric S protein from an attenuated background containing transcription regulating sequences (TRSs) duplication and the introduction of restriction sites, as described in [46]. Engineered restriction sites are in italics, duplicated sequences (Dup. Seq.) into grey boxes. Each gene is in a color box as in the upper virus genome scheme, with stop and ATG codons indicated.

**Figure 2:**
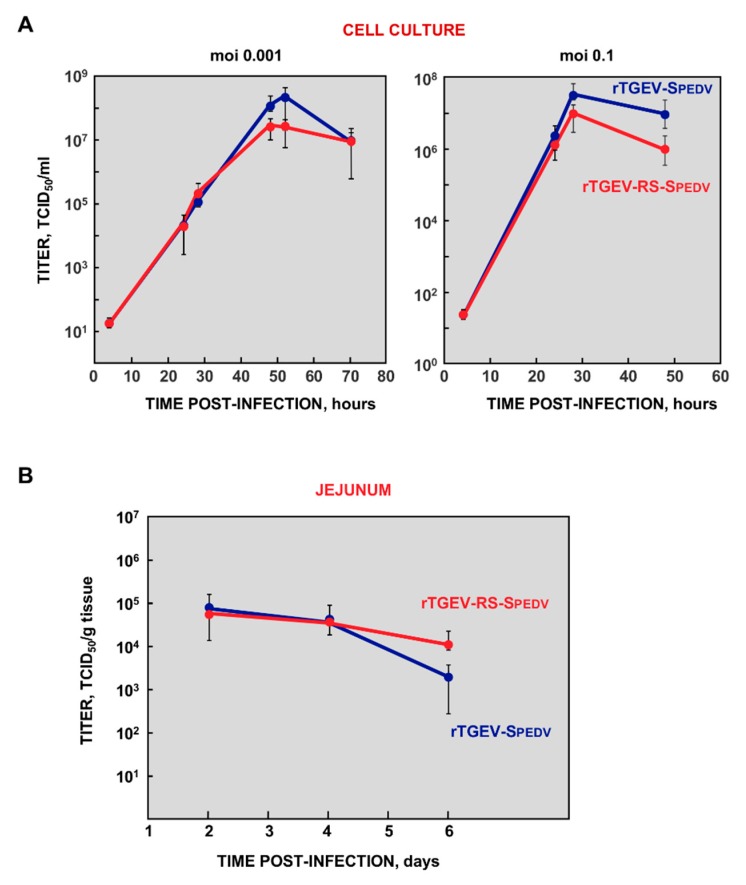
Growth kinetics of rTGEV-SPEDV viruses. (**A**) Viral growth in cell cultures. Vero cells were infected with rTGEV-SPEDV (blue) or rTGEV-RS-SPEDV (red) viruses at MOI of 0.001 (left panel) or 0.1 (right panel). Aliquots of culture supernatants were recovered at the indicated times post-infection, and virus titers analyzed. The values represent viral titers from three independent experiments. (**B**) Replication of rTGEV-SPEDV viruses in enteric tract. Five-day-old piglets were orally inoculated with 10^6^ TCID_50_/piglet. Jejunum samples were taken at the indicated times post-infection, and virus present in the samples was titrated. The values represent viral titers from three different animals. Error bars represent the standard deviation for each value.

A chimeric S protein (SPEDV-TGEV) was designed. The N terminus of the protein contained the exposed domain of PEDV S protein from a virulent US strain (Figure 1A), including the epitopes recognized by neutralizing antibodies, and the heptad-repeats domains (aa 1 to 1325). The C-terminus of the protein was that from the enteropathogenic TGEV SC11 virus [36], including the transmembrane domain and C-terminus (aa 1389 to 1448). The chimeric protein was cloned in the TGEV infectious cDNA [30], replacing TGEV S protein (rTGEV-SPEDV, Figure 1B). In addition, chimeric SPEDV-TGEV protein was cloned in an infectious cDNA from an attenuated TGEV, which contained duplications of transcription regulating sequences (TRSs) and engineered unique restrictions sites (RS) to avoid the overlapping of consecutive genes [46] (rTGEV-RS-SPEDV, Figure 1C). Recombinant viruses rTGEV-SPEDV and rTGEV-RS-SPEDV were recovered after transfection of Vero cells with the wild-type and attenuated cDNAs, respectively. These viruses required trypsin to efficiently infect Vero cells, and form syncytia, similarly to PEDV virus. Growth kinetics in cell culture was analyzed by infecting Vero cells at two multiplicities of infection (MOI)moi of 0.1 and 0.001. Both viruses reached peak titers at 28 or 48 hpi, depending on the MOI (Figure 2A). Nevertheless, rTGEV-RS-SPEDV virus peak titers were five- to 10-fold lower than those for parental rTGEV-SPEDV virus (Figure 2A).

### 3.2. Virulence of Chimeric Viruses

**Figure 3:**
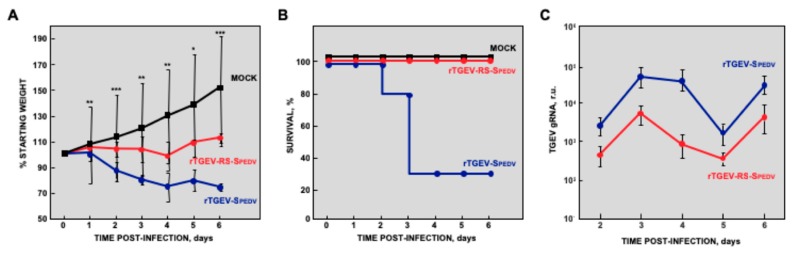
Virulence of rTGEV-SPEDV viruses in suckling piglets. Five-day-old piglets were orally inoculated with 10^6^ TCID_50_/piglet. Animals were mock infected (mock, black) or infected with rTGEV-SPEDV (blue) or rTGEV-RS-SPEDV (red) viruses. Clinical signs were observed daily. Weight loss (**A**) and survival (**B**) are represented. The values are the mean from nine independent animals. (**C**) Virus load in feces was evaluated by RT-qPCR. Error bars represent the standard deviation for each value; *p* values, * <0.05, ** <0.01, *** <0.001.

To analyze the virulence of control rTGEV-SPEDV and attenuated rTGEV-RS-SPEDV viruses, five-day-old piglets were orally inoculated with 10^6^ TCID_50_/animal. Interestingly, despite the difference in viral titers observed in cell cultures, viral titers in the jejunum of infected piglets were similar for both viruses at 2 and 4 dpi (Figure 2B). At 6 dpi, rTGEV-SPEDV titers were slightly lower than those for rTGEV-RS-SPEDV virus, suggesting a decreased number of gut epithelial cells available for reinfection. Piglets inoculated with parental rTGEV-SPEDV virus lost weight after infection (Figure 3A) and 70% died (Figure 3B). This data indicates that the chimeric rTGEV-SPEDV control virus was virulent. In contrast, animals infected with rTGEV-RS-SPEDV virus maintained weight (Figure 3A) and all of them survived (Figure 3B), indicating that the virus was attenuated. Interestingly, virus shedding in the feces of piglets infected with attenuated rTGEV-RS-SPEDV virus was up to 50-fold lower than that observed in the animals infected with the virulent parental rTGEV-SPEDV virus (Figure 3C).

**Figure 4:**
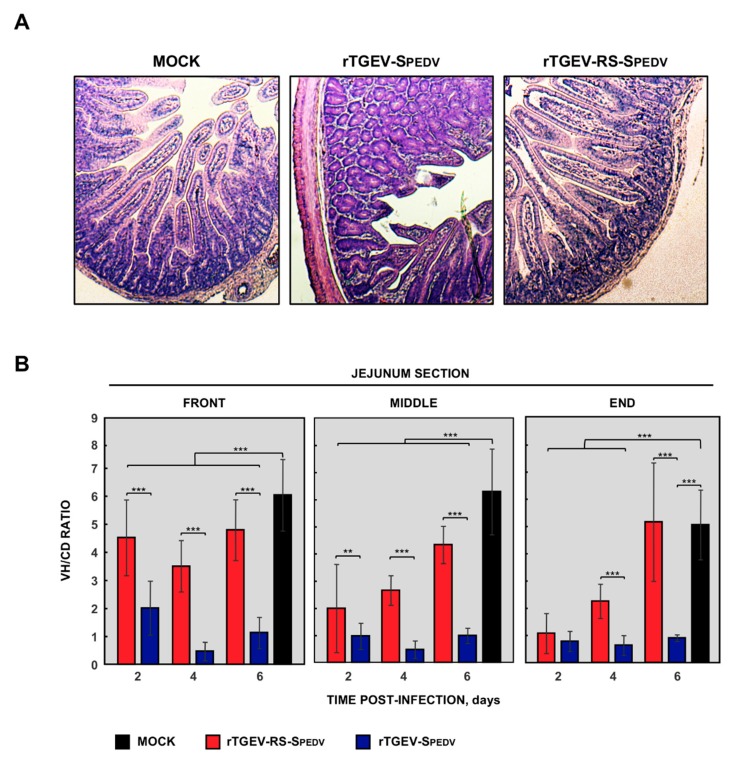
Intestinal damage in suckling piglets infected with rTGEV-SPEDV viruses. (**A**) Representative hematoxylin-eosin-stained front sections from non-infected animals (mock), or animals infected with rTGEV-SPEDV or rTGEV-RS-SPEDV viruses, at six days post-infection. Pictures were obtained with a 2.5× objective. (**B**) Villous height to crypt depth (VH/CD) ratio in front, middle, and end sections at different days post-infection. Non-infected animals (black), or animals infected with rTGEV-SPEDV (blue) or rTGEV-RS-SPEDV (red) viruses. Represented values are the mean from three independent animals, two fields per section. Error bars represent the standard deviation for each value; *p* values, ** <0.01, *** <0.001.

In agreement with the observed clinical signs, epithelial degeneration and exfoliation in the different parts of the jejunum was observed in animals infected with the virulent rTGEV-SPEDV virus, accompanied by inflammatory infiltration and shortening of the villi (Figure 4A). Interestingly, the jejunum damage was significantly lower in animals infected with the attenuated rTGEV-RS-SPEDV virus (Figure 4B). Altogether, the data indicate that rTGEV-RS-SPEDV virus was partially attenuated.

**Figure 5:**
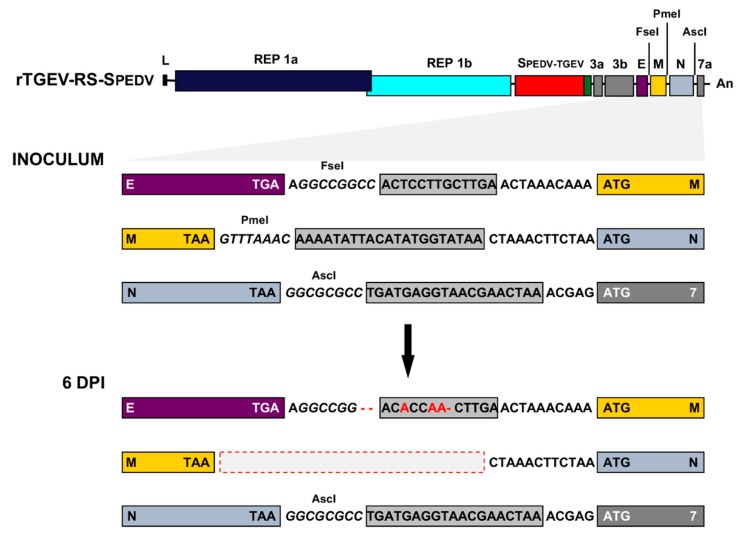
Genetic stability of rTGEV-RS-SPEDV vaccine candidate. Five-day-old piglets were orally inoculated with 10^6^ TCID_50_/piglet. Jejunum samples were taken at 2, 4, and 6 days post-infection. RNA from these samples was extracted, virus was detected by RT-PCR, and the detected bands were sequenced. The modifications introduced in the recombinant virus used to inoculate the piglets (inoculum) were represented as in Figure 1C. The sequence changes observed at day 6 post-infection are represented in red: Dashes indicate nucleotide deletions, red nucleotides represent point mutations, and dashed box indicates large nucleotide deletion.

The main concern for the use of modified live vaccines based on attenuated viruses is their biosafety. Reversion to virulence or recombination with circulating strains is in the basis for the emergence of novel virulent PEDV strains, especially in Asia [29,56]. The rTGEV-RS-SPEDV virus genetic stability, which may influence its safety as vaccine candidate, was evaluated in cell culture. After 15 passages in cell culture, viral genome region comprising from 3b gene to 3′UTR (nucleotides 25856 to 28544) was sequenced. No changes appeared in the passed virus compared with the parental one, strongly suggesting that the engineered virus was genetically stable in cell culture. To address virus stability in vivo, the 3′-ends (nt 25856 to 28544) of rTGEV-SPEDV and rTGEV-RS-SPEDV viruses were sequenced, using RNA isolated from the jejunum of five-day-old infected piglets at six days post-infection. No modifications were found in the parental rTGEV-SPEDV virus. In the rTGEV-RS-SPEDV virus, modifications were only detected in the engineered TRS duplicated sequences (Figure 5). Small deletions and point mutations were found between E and M genes, eliminating the engineered FseI restriction site, but maintaining duplicated sequence (Figure 5). Extensive deletion was observed between M and N genes, reverting the sequence to that of the wild-type virus (Figure 5). No changes were observed in the engineered mutations between N and 7 genes (Figure 5). Interestingly, when viral RNA was isolated from feces of 21-day-old piglets at seven days post-vaccination (see below) and rTGEV-RS-SPEDV virus was sequenced, the same modifications were observed. This data strongly suggests that the rTGEV-RS-SPEDV genome recovered from piglets may represent the in vivo evolution of the engineered virus. It is worth noting that TGEV attenuation was similar with the three TRSs duplications and with just the duplication between N and 7 genes [46], suggesting that in vivo evolved rTGEV-RS-SPEDV virus may still be attenuated.

### 3.3. Protection Conferred by rTGEV-RS-SPEDV Virus

**Figure 6:**
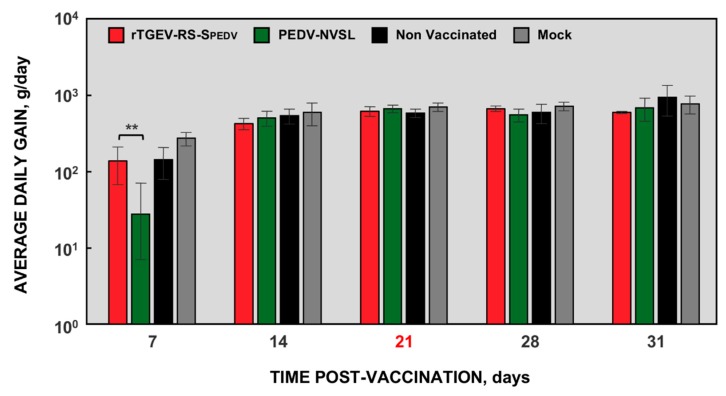
Weight gain in vaccinated animals. Three-week-old pigs were divided into four groups. Animals from two groups were vaccinated with 10^6^ TCID_50_/animal of rTGEV-RS-SPEDV virus (red) or a virulent US PEDV strain (PEDV-NVSL, green). Three weeks after vaccination (21 dpv indicated in red), non-vaccinated animals were either mock infected (Mock, grey) or challenged with 10^7^ TCID_50_/animal of a virulent PEDV strain (Non Vaccinated, black). Vaccinated animals were also challenged with the same dose as non-vaccinated animals. Clinical signs and weight were observed daily after vaccination and challenge, and once per week during the course of the experiment. Average daily gain represents the mean from six different animals. Error bars represent the standard deviation for each value; *p* value, ** <0.01.

PEDV infects animals of all ages, although the severity of the clinical signs is age-dependent [57]. Pregnant sows are the most suitable models to evaluate the effect of PEDV vaccine candidates, as the target animals are neonatal piglets, and lactogenic immunity has an important role in protection [26]. Nevertheless, availability, cost, and housing resources limited its use in preliminary vaccine candidate trials [16,18], and a young pig model has been proposed for preliminary vaccine efficacy trials [13,16]. Therefore, to evaluate the protection conferred by rTGEV-RS-SPEDV virus, 21-day-old pigs were used. Group 1 (G1) was vaccinated with rTGEV-RS-SPEDV virus, group 2 (G2) was vaccinated with a virulent US PEDV strain (PEDV-NVSL), and two groups of animals (G3 and G4) were not vaccinated (Table 1). Twenty-one days after vaccination, animals from G1, G2, and G3 were challenged with a virulent recombinant PEDV strain (rPEDV) (Table 1). As expected, due to the animal age, no clinical sings of enteric disease such as diarrhea, vomiting, or dehydration were observed after vaccination or challenge (data not shown). Nevertheless, animals vaccinated with the virulent PEDV-NVSL strain showed a delay in weight gain the first week after vaccination, compared with non-infected animals or those vaccinated with rTGEV-RS-SPEDV virus (Figure 6). Similar observations were reported for other virulent US isolates [13]. These data indicate that rTGEV-RS-SPEDV virus was also attenuated in 21-day-old piglets.

**Figure 7:**
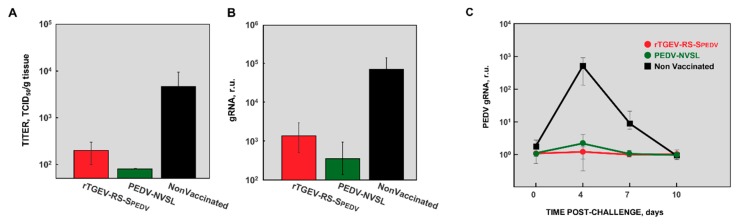
PEDV titers in the enteric tract of animals vaccinated and challenged with virulent PEDV. Four days after the challenge with a virulent PEDV strain, jejunum samples were collected from three piglets per group. Virus presence in these samples was analyzed by titration in Vero cells (**A**) or by RT-qPCR (**B**). In addition, PEDV challenge virus shedding in fecal samples was evaluated at different days after challenge, by RT-qPCR (**C**). Animals vaccinated with rTGEV-RS-SPEDV virus (red); pigs vaccinated with the virulent US PEDV-NVSL strain (green); non-vaccinated and challenged animals (black). Error bars represent the standard deviation for each value.

Virus titers were evaluated in the jejunum of challenged animals. The challenge virus replicated efficiently in the gut of non-vaccinated animals (Figure 7A). In contrast, the titers were reduced more than 25-fold or 50-fold in animals vaccinated with rTGEV-RS-SPEDV and PEDV-NVSL viruses, respectively (Figure 7A). Similar results were obtained when viral RNA presence was evaluated, with up to 200-fold reduction in the accumulation of PEDV RNA (Figure 7B). Interestingly, after challenge, PEDV virus was only shed by non-vaccinated animals (Figure 7C). Altogether, these data indicate that the challenge virus did not efficiently infect the vaccinated animals. Furthermore, these results indicate that rTGEV-RS-SPEDV protected the animals against virulent PEDV challenge.

### 3.4. Humoral Response Elicited by rTGEV-RS-SPEDV Virus

**Figure 8:**
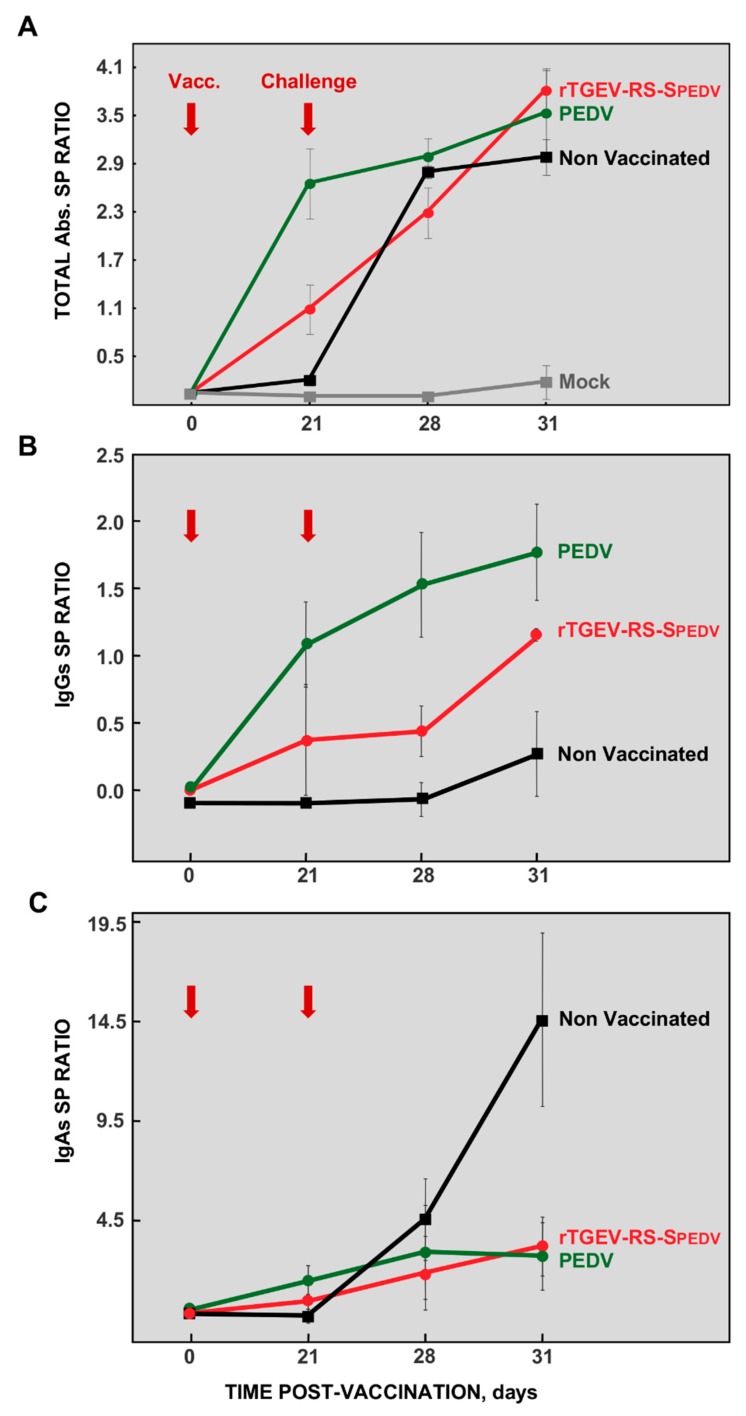
Humoral response induced in vaccinated animals. Serum samples from vaccinated and non-vaccinated animals were collected at the indicated times post-vaccination. Vaccination day (Vacc.) and challenge day were indicated by red arrows. Antibodies specific for PEDV were analyzed by ELISA, measuring total antibodies (**A**), IgGs (**B**) and IgAs (**C**). Mock, non-vaccinated and non-challenged animals (grey); non-vaccinated and challenged animals (black); animals vaccinated with the virulent PEDV-NVSL strain (green) or with the vaccine candidate rTGEV-RS-SPEDV (red). The values represent the mean from, at least, three different animals. Error bars represent the standard deviation for each value.

The antibody response specific for PEDV was evaluated in the sera from vaccinated and non-vaccinated animals. Before challenge (21 dpv), vaccination elicited PEDV-specific antibodies to a higher level in PEDV-NVSL vaccinated animals than in rTGEV-RS-SPEDV vaccinated ones (Figure 8A). This result was expected, as the ELISA test was developed against the whole PEDV virus, and the chimeric rTGEV-RS-SPEDV virus only expressed PEDV S protein. After challenge at 28 and 31 dpv, PEDV total antibodies increased in all challenged groups, as expected. Interestingly, the increase was slower in the vaccinated pigs, in agreement with a decreased challenge virus replication in these animals. Total anti-TGEV antibodies were also evaluated by ELISA. All animals were seronegative at the time of vaccination. When challenge was performed, the level of TGEV antibodies was not significant in any group, and the all the animals remained seronegative after PEDV challenge. This result was expected, as S protein is the main inducer of antibodies against TGEV and it was not present in any of the viruses used for vaccination or challenge. In addition, it has been shown that there is no cross-reactivity between TGEV and PEDV [58].

To further analyze the humoral response specific for PEDV, the levels of PEDV-specific IgGs (Figure 8B) and IgAs (Figure 8C) were evaluated. The data were similar to those obtained for total anti-PEDV antibodies. It has been demonstrated that protection against PEDV infection correlates with IgA levels [21,44,59,60]. It is worth noting that IgA levels in the sera of vaccinated animals, after challenge, were significantly reduced compared with non-vaccinated animals (Figure 8C), strongly suggesting that challenge virus did not elicit a potent IgA response in the gut of vaccinated animals, most likely due to reduced replication in the enteric tract, correlating with protection.

**Figure 9:**
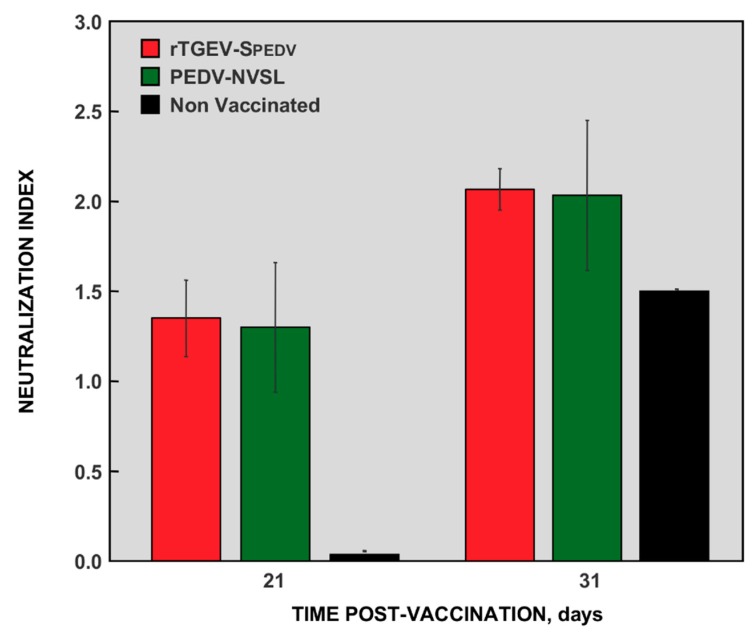
Neutralizing antibodies produced in vaccinated animals. Virus neutralization assay was performed using serum samples, collected before (21 dpv) or after (31 dpv) challenge, from rTGEV-RS-SPEDV virus (red) or PEDV-NVSL (green) vaccinated animals, and from non-vaccinated and challenged animals (black). The values represent neutralization index from three different animals. Error bars represent the standard deviation for each value.

The neutralization capability of the antibodies elicited was evaluated. As expected, non-vaccinated animals only induced the production of neutralizing antibodies after challenge (Figure 9). In contrast, pigs vaccinated with both PEDV-NVSL and rTGEV-RS-SPEDV virus induced a significant level of neutralizing antibodies before challenge, which were slightly increased after challenge (Figure 9).

Altogether, the data indicate that rTGEV-RS-SPEDV virus conferred protection against PEDV infection in the young pig model and could be the basis for an effective vaccine candidate.

## 4. Discussion

An attenuated chimeric rTGEV virus expressing the ectodomain of a virulent US PEDV S protein (rTGEV-RS-SPEDV) was engineered as vaccine candidate for PEDV and evaluated in a young piglet model system. The rTGEV-RS-SPEDV virus elicited a neutralizing humoral response specific for PEDV. In fact, vaccinated animals were protected against challenge with a virulent PEDV virus.

The attenuated live vaccine candidates are the best option for PEDV vaccination because non-replicative antigens, as in inactivated vaccines, do not induce mucosal immunity and protection [16,26]. Traditionally, attenuated live vaccines were developed for classical PEDV strains, consisting of serial passages of the virulent virus in cell culture, as tissue culture adaptation led to attenuation in vivo. These vaccines were not effective to control the infection, as they were extensively used in countries were novel PEDV strains emerged [14,15]. As a consequence, many groups have developed traditionally attenuated live vaccines based on novel epidemic PEDV strains. In general, it has been shown that more than 100 passages of the virulent virus in cell culture are required to attenuate PEDV in vivo [61,62,63,64,65,66].

It is worth noting that in our rTGEV-RS-SPEDV vaccine candidate, the attenuating mutations were specifically designed and introduced in several locations of the viral genome. In contrast, traditionally attenuated vaccines contain non-controlled mutations throughout the genome introduced during passages in cell cultures that, after passages in vivo, may lead to vaccine virus reversion to virulence.

The engineered rTGEV-RS-SPEDV vaccine candidate has the advantage of replicating in the jejunum of infected piglets as efficiently as a virulent control virus, without causing significant pathology, and most likely leading to a protective IgA response. Indeed, vaccinated animals were protected against virulent PEDV challenge. In contrast, traditionally attenuated vaccines, as a consequence of adaptation to cell culture, have a dramatically reduced replication in the enteric tract [66] and, as a consequence, these vaccines do not actively replicate to induce good IgA and lactogenic immune responses [1,13,26].

Recently, a swine enteric coronavirus (SeCoV) that is a recombinant between TGEV and PEDV has been described in Europe [67,68,69]. The SeCoV contains a genome background identical to TGEV, but expresses the S protein from PEDV. In addition, recombination led to extensive deletions in the coding sequence and in TRS of TGEV gene 3a, and, as a consequence, protein 3a may not be expressed by SeCoV. It is worth noting that SeCoV was isolated from diarrheic samples in all cases, suggesting that it is virulent. The engineered parental rTGEV-SPEDV virus resembles the recombinant SeCoV and, as shown by our data, is virulent in suckling piglets. In contrast, the engineered virus (rTGEV-RS-SPEDV) used as a vaccine candidate was attenuated. There are some genetic differences that may eventually allow the discrimination between SeCoV and the rTGEV-RS-SPEDV vaccine candidate as (i) rTGEV-RS-SPEDV virus did not contain the full PEDV S gene sequence, (ii) it expressed TGEV protein 3a, and (iii) because of the TGEV strain used to engineer the infectious cDNA, it did not express TGEV protein 3b [70].

The rTGEV-RS-SPEDV vaccine candidate was genetically stable in tissue culture; nevertheless, it evolved in vivo. Therefore, its biosafety could be improved by adding an additional safety guard at the 5′-end of PEDV virus genome (in the replicase gene), at a distal position from the attenuating mutations introduced by duplicating sequences closed to M, N and 7a genes (Figure 1) [71,72,73,74]. The optimized vaccine candidate should keep a balance between virus replication attenuation and eliciting an efficient immune response.

## Figures and Tables

**Table 1:** Animal groups in the protection experiment; 21-day old piglets were randomly assigned to four different groups.

Group	Vaccination ^1^	Challenge ^2^
1	rTGEV-RS-SPEDV	rPEDV
2	PEDV-NVSL	rPEDV
3	Mock	rPEDV
4	Mock	Mock

^1^ Oral inoculation of 10^6^ TCID_50_/pig; ^2^ Twenty-one days post-vaccination. Oral inoculation of 10^7^ TCID_50_/pig.

**Table 2:** Oligonucleotides used for virus sequencing. PCRs covering different transmissible gastroenteritis virus (TGEV) regions, including transcription regulating sequences (TRSs), duplications were performed.

Forward	Reverse	Size (bp)
5′-TATGACGTTTCCTAG-3′	5′-TTACATTAATGTGACTAAC-3′	1783
5′-TGTGAATGACCTCACGTTGC-3′	5′-TAGATTGAGAGCGTGACCTTG-3′	2452
5′-CGCTTGGTAGTCGTGGTGC-3′	5′-ACATTTTAAACAATCACTAG-3′	1252
